# Formal and informal digital health in Tanzania: healthcare workforce, regulatory and trust implications of e-patients and virtual care and the development of the TRUST framework

**DOI:** 10.3389/frhs.2026.1859331

**Published:** 2026-06-30

**Authors:** Kahabi Ganka Isangula, Judith Keebisilo Leo, James Tumaini Kengia

**Affiliations:** 1 School of Nursing and Midwifery, Aga Khan University, Dar Es Salaam, Tanzania; 2 School of Computational and Communication Science and Engineering, The Nelson Mandela African Institution of Science and Technology (NM-AIST), Arusha, Tanzania; 3 Prime Minister’s Office, Regional Administration and Local Government (PMO-RALG), Dodoma, Tanzania

**Keywords:** digital health, digital health governance, e-patients, Tanzania, trust, trust framework, virtual care, workforce preparedness

## Abstract

Digital health and virtual care are increasingly reshaping how healthcare is organized, delivered, and experienced. In Tanzania, digital transformation is evident in national commitments to digital health governance, health information exchange, hospital information systems, digital referral and emergency transportation, telemedicine, and emerging diagnostic applications supported by artificial intelligence. At the same time, patients are increasingly using social media, mobile platforms, and other digital channels to seek health information, consultations, diagnoses, and medicines, creating new forms of digitally mediated care that often operate alongside formal healthcare systems. Using a narrative synthesis design informed by a practice-based perspective, this study reviewed policy documents, national strategies, implementation reports, peer-reviewed empirical studies, global guidance documents, and conceptual literature relevant to Tanzania's digital health ecosystem. Evidence was identified through a structured search, source-selection, and thematic synthesis process. The review examined three interrelated questions: (i) how Tanzania's digital health and virtual care landscape is evolving; (ii) how e-patient behaviours, informal digital consultations, and emerging e-provider practices are interacting with formal health systems; and (iii) what these developments imply for workforce preparedness, clinical governance, patient safety, antimicrobial stewardship, confidentiality, accountability, and trust. The findings suggest that Tanzania's digital health transition is not primarily a technology-adoption challenge but a practice challenge involving how healthcare workers, patients, digital technologies, and governance systems interact in everyday care. While substantial progress has been made in establishing digital health policies and governance frameworks, important gaps remain in workforce preparedness, digital professionalism, virtual care governance, stewardship, privacy protection, and management of informal digital health practices. The review highlights the growing influence of e-patients and digitally mediated health-seeking behaviours in reshaping traditional patient–provider relationships and expectations of care. To support policy and implementation action, this paper proposes the TRUST framework: **T**ransparent Credentials and Workforce Preparedness, **R**obust Clinical Governance, **U**ser-Centred Engagement, **S**afety and Stewardship, and **T**rusted e-Health Technologies and Data Practices. Grounded in a practice-based perspective and implementation evidence, TRUST is proposed as a practice-based framework for strengthening accountable digital health work practices, patient trust, workforce readiness, and regulatory preparedness in Tanzania and similar settings where formal and informal digital health pathways increasingly coexist.

## 1 Introduction

Digital technologies are changing health systems in two ways at the same time. On one side, they enable governments, healthcare institutions and healthcare workers to better record care and reduce errors during documentation, hence enhancing data quality, tracking patients, managing commodities, supporting decisions, and strengthening coordination across levels of the system (1, 2). On the other side, they also change how people understand illness, search for advice, and judge the credibility of health workers and provide feedback (1, 2). This double transformation matters because healthcare is not only technical but also, depends on therapeutic relationships, confidentiality, fairness, legitimacy, and trust.

Globally, the World Health Organization (WHO) has emphasized that digital transformation can strengthen health systems only when it is strategically governed, integrated into health financing and human resources, and designed for safe and equitable use rather than simply converting paper systems into electronic platforms (1, 2). Consequently, the competencies required of healthcare workers increasingly extend beyond clinical skills to include digital competence, meaning the ability to use digital systems safely, communicate effectively through virtual channels, interpret digitally supported decisions responsibly, and maintain patient confidence in technology-mediated care (3, 4). Strengthening these competencies is essential for ensuring that digital technologies are effectively leveraged to improve the timely availability, accessibility, and use of high-quality health information, ultimately enhancing service delivery and continuity of care (3).

Tanzania's digital health agenda has evolved progressively from early direction-setting strategies to more coordinated national governance mechanisms. The National eHealth Strategy (2013–2018) outlined a vision for safer, higher-quality, and better-connected health services supported by information and communication technologies (3). Tanzania subsequently adopted the National Digital Health Strategy (2019–2024) to respond to the growing reality of multiple digital systems operating in parallel with limited coordination and weak interoperability (4). The strategy emphasizes improved coordination, interoperability, and governance while recognizing that digital information sharing must comply with standards for safety, security, confidentiality, and privacy.

A key message from recent Tanzanian digital health governance efforts is that fragmentation is not a simply a technical issue. Instead, fragmented digital systems affect workload, efficiency, data quality, continuity of care, and accountability (4, 5). To partly address fragmentation, Tanzania has therefore invested in a broader “architecture” approach to integration of digital health solutions. The Tanzania Health Enterprise Architecture is presented as a national blueprint for aligning health sector strategies and operations with digital health solutions while reducing duplication and lack of interoperability (5). Similarly, the Tanzania Digital Health Investment Road Map (2017–2023) was developed to link improvements in data systems and data use to better service delivery, better tracking of clients, and more efficient allocation of resources (6). At the service delivery level, the impact of these policies is increasingly visible. Tanzania has expanded the use of national and facility systems such as the District Health Information Software Version 2 (DHIS2) reporting platforms and the Government of Tanzania Health Operations Management Information System (GoTHOMIS). In parallel, Tanzania has advanced interoperability through a national health information exchange approach. A major national partnership implemented between 2014 and 2019 reported that Tanzania implemented an interoperability layer enabling data exchange across multiple separate systems, contributing to improved data availability and time savings, with the system incorporated into national strategy and managed by Tanzanian officials (7).

However, formal digital systems represent only one dimension of digital transformation. Everyday digital practices among patients and communities are also reshaping healthcare-seeking behaviours. The concept of “Medicine 2.0” partly helps explain why patients are no longer only passive recipients of information from healthcare workers (8). Patients participate, share, and seek advice through digital networks (8, 9). Evidence from different settings shows that online health information can influence decision making in both helpful and harmful ways, and the quality of the patient–provider relationship can shape whether online searching increases engagement or increases mistrust (10). Tanzania-specific analyses have further highlighted the rise of e-patients and the expansion of informal or insufficiently regulated digital consultations (the informal e-providers) accessed through mobile applications and social media platforms raising concerns related to safety, professional accountability, and equity (11).These developments are occurring within a context where confidentiality and patient safety are increasingly under pressure. Tanzania adopted a national data protection law (Personal Data Protection Act, 2022; English publication 13 June 2023) that establishes principles for personal data protection and an oversight structure, including the establishment of a Personal Data Protection Commission (12, 14). Nevertheless, everyday virtual care practices may still expose patients to privacy risks. This occurs through screenshots, shared devices, unsecured messaging applications, unclear data-storage arrangements, and inconsistent documentation practices if systems and healthcare workers are insufficiently prepared (14). Safety concerns also extend beyond privacy into medication use and antimicrobial stewardship. Tanzania continues to face challenges related to non-prescription antibiotic dispensing and antimicrobial self-medication, both recognized drivers of antimicrobial resistance (AMR) (14, 15). Although the country has responded through national AMR action plans aligned with global priorities (14–15), the rapid growth of informal digital consultations (e-providers) and online medicine access could create additional channels for inappropriate antimicrobial advice or medicine access if regulatory and workforce safeguards are not strengthened. However, direct Tanzanian evidence linking informal digital consultations to AMR outcomes remains limited and should be investigated.

The problem addressed in this article is the growing mismatch between Tanzania's formally stated digital health policies, governance procedures, professional accountability arrangements, and the actual digital health practices emerging on the ground. While the formal health system is moving toward regulated, interoperable, and institutionally governed digital platforms, patients and formal, informal, or insufficiently regulated providers are increasingly using social media, mobile messaging, online medicine channels, and other digital tools to seek and provide advice, consultation, reassurance, referral, and medicines outside clearly defined systems of credentialing, documentation, privacy protection, referral, and clinical accountability (11). This mismatch creates governance and legitimacy gaps because professional training, curricula, and formal procedures may not fully reflect the emerging realities of e-patient, e-provider, e-consultation, and e-nursing practices that increasingly shape healthcare delivery. The article therefore specifically focuses on the implications of these transformations for workforce preparedness, provider legitimacy, confidentiality, patient safety, antimicrobial stewardship, and trust within digitally mediated healthcare environments.

The manuscript was developed with an understanding that several implementation science and digital health frameworks already provide valuable insights into technology adoption and health-system transformation. Frameworks such as the Consolidated Framework for Implementation Research (CFIR), the Non-adoption, Abandonment, Scale-up, Spread, and Sustainability (NASSS) framework, RE-AIM/PRISM, and the Technology Acceptance Model (TAM) contribute substantially to understanding implementation determinants, sustainability, contextual fit, scale-up challenges, and user acceptance of digital technologies (16–20). However, these frameworks do not, by themselves, provide a Tanzania-focused operational bridge between workforce competences, provider verification, confidentiality, antimicrobial stewardship, regulatory accountability, and patient trust in settings where informal digital consultations and social-media-mediated care practices are rapidly expanding (11). Existing implementation frameworks were also largely developed within high-income health-system contexts and do not explicitly address the coexistence of formal digital infrastructures with informal mobile-phone-mediated care pathways that are increasingly common in many low- and middle-income settings.

In response, the manuscript proposes the Transparent credentials and workforce preparedness, Robust clinical governance, User-centred engagement, Safety and stewardship, and Trusted e-Health technologies and data practices (TRUST) framework as a context-adapted practice -based operational framework to support workforce development, regulatory preparedness, and accountable virtual care implementation in Tanzania and similar African settings. The TRUST framework is not proposed as a substitute for traditional implementation science frameworks (16–20). Instead, it complements these frameworks by addressing a more specific operational and governance problem: how to strengthen workforce behaviour, regulatory accountability, patient safety, stewardship, confidentiality, and trust within rapidly evolving digital and virtual care African healthcare environments where formal and informal digital health pathways increasingly coexist.

## 2 Conceptual/theoretical framework: a practice-based perspective to digital health work practices

This manuscript is informed by a practice-based perspective, which shifts analytical attention from formal policies, organizational structures, and technological systems to the everyday activities through which healthcare work is enacted. Practice-based scholarship argues that work cannot be fully understood through official procedures, professional roles, or technological designs alone. It emphasizes that knowing, learning, and organizing emerge through situated practices which is the recurrent patterns of action, interaction, interpretation, and problem-solving that individuals perform within particular social, material, and institutional contexts (21–23).

Furthermore, practice theorists view organizations not as static entities governed solely by formal rules but as ongoing accomplishments continuously produced and reproduced through what people do in practice (24, 25). Practices encompass not only observable actions but also the use of tools and technologies, embodied skills, tacit knowledge, professional judgment, emotions, motivations, social relationships, and context-specific understandings that shape how work is conducted (26, 27). From this perspective, there is often a distinction between formal prescriptions of work and the realities of work as performed in everyday settings.

Additionally, the practice lens is particularly relevant for understanding digital health transformation. Digital technologies are frequently introduced through policies, strategic plans, interoperability frameworks, and implementation guidelines that specify how health information should be recorded, shared, analysed, and used. However, evidence from digital health implementation research consistently demonstrates that technologies do not operate independently of human practices (18, 27). Their effects are seen to emerge through interactions among healthcare workers, patients, technologies, organizational routines, institutional expectations, and broader social contexts (17, 28). From a practice perspective, digital technologies are not merely tools that support existing healthcare activities. They actively reshape how healthcare workers communicate, document clinical encounters, exchange information, make decisions, coordinate referrals, prescribe medications, monitor patients, and protect confidentiality. Similarly, digital technologies influence how patients seek information, evaluate health advice, engage with providers, and participate in healthcare decision-making. Consequently, digital transformation involves not only technological change but also the emergence of new forms of work practice, professional interaction, accountability, and patient participation. This viewpoint is particularly important in the Tanzanian context, where rapid digitalization is occurring alongside expanding internet access, mobile-phone penetration, electronic health information systems, telemedicine initiatives, artificial intelligence applications, and social-media-mediated health communication (11). While considerable attention has been given to strengthening digital infrastructure, interoperability, governance frameworks, and health information systems, less attention has been paid to how healthcare workers and patients actually use these technologies in practice and how these practices influence safety, trust, quality of care, and professional accountability.

The practice lens also provides a useful framework for understanding the emergence of digitally mediated forms of care that increasingly operate alongside formal health systems. These include e-patients seeking health information online, informal digital consultations through social media and messaging platforms, e-consultations between providers and patients, remote mentoring and tele-support arrangements, and emerging forms of e-nursing and digitally enabled care coordination. Rather than viewing these developments simply as technological innovations or deviations from formal healthcare systems, practice theory encourages examination of how they become normalized through everyday interactions and how they reshape relationships between patients, healthcare workers, technologies, and institutions (8, 11).

A practice-based perspective also highlights an important challenge for workforce preparedness. Professional education and training often focus on formal competencies, technical skills, and regulatory requirements. However, healthcare workers increasingly operate in environments where digital technologies introduce new forms of communication, decision-making, information sharing, patient engagement, and ethical responsibility. Competence therefore extends beyond technical proficiency to include the ability to navigate complex digital work practices, manage evolving patient expectations, critically evaluate digital information, maintain confidentiality, exercise professional judgment, and uphold accountability within digitally mediated care environments.

The present review adopts a practice-based perspective to interpret evidence on digital health transformation in Tanzania, examining how emerging digital health practices shape workforce preparedness, governance, patient safety, confidentiality, antimicrobial stewardship, and trust. This is because the insights from practice-based literature suggest that digital transformation should be understood not merely as the deployment of technologies but as the reconfiguration of everyday healthcare practices. This practice-based understanding provides the analytical foundation for the development of the TRUST framework (detailed in subsequent sections). Specifically, it highlights that effective digital health governance requires not only technological infrastructure and regulatory frameworks but also depends on the everyday interactions through which healthcare workers and patients engage with digital technologies and with one another. Informed by these insights, the TRUST framework is conceptualized as a practice-based framework that aligns formal governance structures with the realities of digitally mediated healthcare delivery. A focus on the interaction between policies, technologies, professional practices, and patient behaviours, positions the framework as supporting accountable, safe, and trustworthy digital health implementation in Tanzania and other low- and middle-income country settings undergoing rapid digital transformation. The practice-based literature insights therefore serve as a bridge between the empirical findings presented in the Results section and the development of the TRUST framework.

## 3 Methods

### 3.1 Design

This manuscript is a descriptive, policy and practice-informed narrative synthesis. A narrative synthesis approach was intentionally selected because the evidence base varies from heterogeneous policy documents, implementation reports, empirical studies, governance literature to emerging digital-health practice reports that are not amenable to meta-analytic synthesis. The intent is not to estimate pooled intervention effects, but to support healthcare workers, healthcare service leaders, healthcare training institutions, educators, and regulators with a coherent argument grounded in Tanzania-relevant evidence.

### 3.2 Search approach and selection of sources

A rapid narrative search and evidence-mapping approach was employed to identify policy documents, programme reports, implementation papers, guidance documents, and peer-reviewed literature relevant to digital health transformation, virtual care, workforce preparedness, patient trust, and digital governance in Tanzania. The objective was to generate a policy- and practice-informed synthesis of current developments and emerging challenges rather than conduct a formal systematic review or meta-analysis. Consequently, a broad and iterative search strategy was used to capture diverse forms of evidence reflecting technological, behavioural, regulatory, and health-system dimensions of digital transformation.

### 3.3 Search sources and period

Searches were conducted between January and March 2026 and covered materials published between January 2010 and December 2025, a period corresponding to the accelerated adoption of digital health technologies in Tanzania and globally. Multiple sources were searched to maximize coverage of both peer-reviewed and practice-based evidence, including: PubMed/MEDLINE; Google Scholar; WHO digital health repositories and publications; Tanzanian Ministry of Health and government repositories; national digital health strategy and policy documents; programme and development-partner websites; reports from implementing organizations and professional bodies; and reference lists of eligible articles, reports, and policy documents. Grey literature was intentionally included because many digital health implementation experiences, governance initiatives, and virtual care developments in Tanzania are documented in programme reports, policy documents, technical guidance, and implementation evaluations rather than exclusively in peer-reviewed journals. Scopus and Web of Science were not searched because the review was designed as a rapid narrative synthesis rather than a comprehensive systematic review, and the selected databases and repositories were considered sufficient to capture policy, implementation, governance, and Tanzania-specific digital health literature relevant to the study objectives.

### 3.4 Search strategy

Search terms were combined iteratively using Boolean operators and adapted to the requirements of individual databases and repositories. The search strategy incorporated terms related to digital health systems, patient behaviour, governance, workforce preparedness, safety, and emerging technologies.

Key search concepts included: “*digital health”, “eHealth”, “mHealth”, “virtual care”, “telehealth”, “telemedicine”, “teleconsultation”, “electronic medical record”, “health information management system”, “GoTHOMIS”, “DHIS2”, “health information exchange”, “interoperability”, “enterprise architecture”, “artificial intelligence diagnostics”, “CAD4TB”, “telepathology”, “cough classifier”, “e-patients”, “online health information”, “social media health”, “online medical advice”, “informal digital consultations”, “digital professionalism”, “e-Health literacy”, “patient safety”, “privacy”, “confidentiality”, “data protection”, “misinformation”, “antimicrobial stewardship”, “antimicrobial resistance”, “digital competence”, “health workforce digital skills”, and “Tanzania*”*.*

Additional searches combined these concepts with terms such as “governance”, “regulation”, “trust”, “quality of care”, “health workforce”, and “digital transformation” to identify literature relevant to the article's conceptual focus. Also, terms related to antimicrobial stewardship and antimicrobial resistance were included because one of the study objectives was to examine patient-safety and stewardship implications associated with emerging virtual care practices, informal digital consultations, and online access to medicines.

### 3.5 Eligibility criteria

Sources were included if they:

1. Addressed digital health, virtual care, health information systems, artificial intelligence applications, patient digital behaviour, workforce preparedness, governance, safety, privacy, trust, or antimicrobial stewardship.
2. Focused on Tanzania or contained findings relevant to health-system implementation in Tanzania.
3. Were published in English between January 2010 and December 2025.
4. Included empirical studies, policy documents, implementation reports, programme evaluations, guidance documents, strategy papers, or conceptual analyses relevant to the study objectives; and
5. Contributed evidence addressing at least one of the article's three guiding questions.

Sources were excluded if they:

1. Focused exclusively on technical system design without implications for service delivery, governance, workforce preparedness, or patient behaviour.
2. Were unrelated to health-sector digital transformation.
3. Were duplicate publications.
4. Provided insufficient methodological or contextual information to support interpretation; or
5. Addressed digital technologies outside health care with no relevance to the study objectives.

### 3.6 Source selection

To enhance transparency and methodological rigor, principles adapted from the Preferred Reporting Items for Systematic Reviews and Meta-Analyses (PRISMA) framework were used to guide the identification, screening, and selection of evidence (Table 1). Given the narrative and policy-oriented nature of this review, these principles were applied to support transparent reporting rather than exhaustive evidence retrieval.

**Table 1:** PRISMA-informed source identification and selection.

Component	Description
Review design	Rapid narrative, policy- and practice-informed synthesis
Purpose	To synthesize evidence on digital health transformation, virtual care, workforce preparedness, patient trust, governance, and safety in Tanzania
Search period	January–March 2026
Publication period covered	2010–2025
Information sources	PubMed/MEDLINE, Google Scholar, WHO repositories, Tanzanian government and Ministry of Health repositories, programme and partner organization websites, and reference-list searches
Language	English
Identification	Records identified through database searching (*n* = 214)
	PubMed/MEDLINE (*n* = 68)
	Google Scholar (*n* = 96)
	WHO repositories (*n* = 18)
	Tanzanian Ministry of Health and Government repositories (*n* = 14)
	Programme and partner organization websites (*n* = 10)
	Reference-list searching and additional grey literature (*n* = 8)
Screening	Records after duplicates removed (*n* = 176)
	Records screened by title and abstract/executive summary (*n* = 176)
	Records excluded (*n* = 101)
	Reasons for exclusion:
	Not relevant to Tanzania or comparable sub-Saharan African contexts (*n* = 29)
	Focused only on technical system design without governance/workforce/patient implications (*n* = 24)
	Not related to digital health, virtual care, or patient digital behaviour (*n* = 21)
	Editorials/news items lacking analytical relevance (*n* = 15)
	Duplicate programme descriptions or outdated versions (*n* = 12)
Exclusion criteria	Sources unrelated to health-sector digital transformation, duplicate records, purely technical system descriptions lacking implementation relevance, or sources with insufficient contextual information.
	Full-text sources assessed for eligibility (*n* = 75)
	Full-text sources excluded (*n* = 22)
	Reasons for exclusion:
	Limited relevance to workforce preparedness, trust, confidentiality, stewardship, or governance (*n* = 9)
	Insufficient methodological or contextual detail (*n* = 5)
	Focused exclusively on non-health digital systems (*n* = 4)
	Could not be accessed in full text (*n* = 2)
	Superseded by updated policy/programme documents (*n* = 2)
Inclusion criteria	Sources addressing digital health, virtual care, health information systems, artificial intelligence, patient digital behaviour, governance, workforce preparedness, privacy, trust, patient safety, or antimicrobial stewardship relevant to Tanzania.
	Sources included in narrative synthesis (*n* = 53)
	Included source categories:
	Policy and strategy documents (*n* = 10)
	Laws, governance, and global guidance documents (*n* = 7)
	Empirical quantitative studies (*n* = 14)
	Qualitative and mixed-methods studies (*n* = 8)
	Programme implementation/evaluation reports (*n* = 7)
	Systematic/conceptual reviews and implementation frameworks (*n* = 6)
Source charting variables	Source type, geographical focus, health-system level, digital health domain, implementation relevance, governance implications, workforce implications, patient safety considerations, and key findings
Evidence categorization	National governance, facility-level implementation, community/referral systems, patient-facing digital health behaviours, and global implementation evidence
Quality appraisal	No formal quality appraisal or risk-of-bias assessment conducted
Credibility assessment approach	Source provenance, source categorization, evidence charting, and cautious interpretation according to evidence type and strength
Data management and coding	NVivo 12 (Lumivero)
Synthesis method	Iterative thematic and interpretive synthesis
Analytical outputs	Six thematic domains and development of the TRUST framework
Transparency framework	PRISMA-informed reporting adapted for narrative evidence synthesis

Following the initial search, sources were screened for relevance to the study objectives and organized according to three complementary dimensions: (i) source type, including empirical studies, policy and regulatory documents, programme and implementation reports, global guidance documents, and conceptual or framework papers; (ii) level of health-system relevance, including national governance and policy, facility-level service delivery, community and referral systems, and patient-facing digital health behaviours; and (iii) relevance to the analytical domains of workforce preparedness, clinical governance, confidentiality, patient safety, antimicrobial stewardship, accountability, and trust in digitally mediated care environments.

Sources were prioritized if they directly informed one or more of the study's three guiding questions:

1. *What is changing in Tanzania's digital health and virtual care landscape?*
2. *How are patient behaviours and informal digital care pathways evolving alongside formal health systems?*
3. *What are the implications of these changes for workforce preparedness, confidentiality, patient safety, antimicrobial stewardship, and trust in the health system?*

Where Tanzania-specific evidence was limited, relevant evidence from comparable sub-Saharan African settings and broader global digital health literature was included to provide contextual and interpretive insights. Throughout the review, distinctions were maintained between Tanzania-specific empirical evidence and findings derived from regional or global literature to avoid overgeneralization. A summary of the search process, eligibility criteria, source categories, and synthesis procedures is provided in Table 1.

### 3.7 Source charting

The search yielded approximately 214 records and documents across all sources. After screening for relevance, removal of duplicates, and assessment against the eligibility criteria, fifty-three sources were retained for inclusion in the synthesis.

To support systematic analysis, all included sources were charted using a structured extraction template that captured source characteristics, health-system level, digital health domain, methodological approach, and relevance to the study's analytical focus (Table 2). This process facilitated comparison across diverse forms of evidence and strengthened the transparency of the synthesis.

**Table 2:** Mapping of sources included in the narrative synthesis.

Author/source (Ref No.)	Source type	Health system/analytical level	Relevance to workforce preparedness, trust, safety, and digital health governance
World Health Organization (1)	Global strategy and policy document	Global governance and health-system transformation	Provides the overarching strategic vision for digital health transformation, emphasizing governance, interoperability, workforce capacity, digital literacy, equity, patient-centred care, and trust in digital health systems. Highlights the need for workforce preparedness, leadership, regulatory frameworks, ethical use of digital technologies, data protection, and accountability mechanisms to ensure safe and trustworthy digital health implementation.
World Health Organization (2)	Global normative guideline and evidence-based recommendation document	Health-system implementation and service-delivery level	Provides evidence-informed recommendations on the use of digital interventions for health system strengthening, including client communication, provider decision support, telemedicine, digital health records, referral coordination, and workforce support.
United Republic of Tanzania (3)	National policy/strategy	National governance	Establishes Tanzania's foundational direction for eHealth development, workforce preparedness, and ICT-enabled health services
United Republic of Tanzania (4)	National policy/strategy	National governance	Emphasizes interoperability, governance, confidentiality, digital competencies, and coordinated digital health scale-up
URT & PATH (5)	Enterprise architecture framework	National governance	Provides architectural blueprint for integrated digital health ecosystems and accountability mechanisms
URT & PATH (6)	Digital investment roadmap	National governance	Guides digital health investments, workforce strengthening, interoperability, and service-delivery improvement
Nsaghurwe et al. (7)	Empirical implementation report	National governance/interoperability	Demonstrates implementation of national health information exchange and governance structures supporting safe data sharing
Eysenbach (8)	Conceptual framework	Global health systems context	Introduces Medicine 2.0 and explains changing patient participation, online engagement, and implications for clinician trust
Van De Belt et al. (9)	Systematic conceptual review	Global health systems context	Clarifies definitions of Health 2.0 and Medicine 2.0 relevant to digital patient participation and evolving therapeutic relationships
Thapa et al. (10)	Systematic review	Global/patient behaviour context	Demonstrates how online health information influences patient decision-making and clinician–patient relationships
Isangula (11)	Narrative review/commentary	Tanzania health systems context	Describes emergence of e-patients and informal digital consultations raising concerns regarding accountability and patient safety
Norman & Skinner (12, 13)	Conceptual framework/eHealth literacy	Global patient behaviour context	Introduces eHealth literacy concepts relevant to patient capacity to interpret online health information safely
United Republic of Tanzania (14)	National law/policy	National governance	Establishes legal framework for personal data protection, privacy, and digital confidentiality
WHO (15)	Global policy/guidance	Global governance	Guides antimicrobial stewardship and safe prescribing practices relevant to virtual care environments
URT (15)	National AMR action plan	National governance	Aligns Tanzania with global AMR prevention and stewardship priorities
Damschroder et al. (16)	Implementation science framework	Global implementation science	Introduces CFIR for understanding multilevel determinants of implementation success
Greenhalgh et al. (17)	Implementation science framework	Global digital health implementation	Develops NASSS framework for technology adoption, scale-up, sustainability, and abandonment
Glasgow et al. (18)	Implementation evaluation framework	Global implementation science	Introduces RE-AIM framework for evaluating public health intervention impact and sustainability
Feldstein & Glasgow (19)	Implementation framework	Global implementation science	Develops PRISM framework linking implementation, sustainability, and contextual fit
Davis (20)	Technology acceptance framework	Global technology adoption	Explains technology adoption through perceived usefulness and ease of use
PMO-RALG (29)	System/program documentation	Facility service delivery	Describes GoTHOMIS implementation supporting digital clinical workflows and health service management
PO-RALG (30)	Operational manual	Facility service delivery	Provides practical guidance for GoTHOMIS implementation and workforce system use
Laitinen et al. (31)	Programme evaluation/report	Facility service delivery	Assesses healthcare workers' experiences with hospital information systems and training needs
Laitinen et al. (32)	Empirical study	Facility service delivery	Identifies gaps in healthcare workers' eHealth competencies and digital preparedness
Mwogosi (33)	Systematic review	National/regional health systems	Reviews telemedicine implementation barriers including governance, financing, infrastructure, and workforce capacity
Rumisha et al. (34)	Empirical study	Facility and district systems	Examines HMIS data quality challenges affecting decision-making and continuity of care
Mboera et al. (35)	Empirical study	Facility and district systems	Investigates data utilization, workforce training gaps, and implementation barriers within HMIS
Cronin et al. (36)	Qualitative empirical study	Facility service delivery	Explores staff experiences with hybrid paper–digital systems and workflow inefficiencies
Breuninger et al. (37)	Empirical validation study	Facility diagnostics	Validates AI-supported TB diagnostic tools and highlights interpretive workforce demands
Mremi et al. (38)	Empirical implementation study	Facility diagnostics	Demonstrates telepathology implementation and infrastructure requirements for digital pathology services
WHO (39)	Global ethical guidance	Global governance/AI	Provides ethical and governance guidance for safe AI integration in health systems
Isangula & Haule (40)	Diagnostic protocol study	Community/diagnostic innovation	Introduces AI-supported cough classifier requiring digital diagnostic interpretation competencies
UKRI (41)	Research registry/programme report	Research innovation context	Documents implementation of AI-supported respiratory diagnostics in Tanzania
Dodoo et al. (42)	Systematic review	Sub-Saharan African health systems	Identifies telemedicine implementation barriers including workforce, policy, and infrastructure limitations
WHO (43)	Global implementation guide	Global health systems	Provides operational guidance for telemedicine implementation, governance, and evaluation
Adebayo et al. (44)	Empirical study	Facility virtual care services	Documents teleconsultation trends and sustainability challenges during COVID-19
Miyashita et al. (45)	Qualitative empirical study	Community and patient behaviour	Demonstrates the role of trust, legitimacy, and community relationships in digital health uptake
Alidina et al. (46)	Mixed-methods empirical study	Facility workforce development	Evaluates digital mentorship and tele-support interventions for workforce strengthening
Dillip et al. (47)	Programme implementation study	Community-referral systems	Demonstrates digitally supported referral and continuity-of-care systems linking community and facility care
Njiro et al. (48)	Programme evaluation	Community emergency systems	Evaluates digital coordination of emergency maternal transportation systems
Munishi et al. (49)	Programme implementation study	Community emergency systems	Describes community-based digital transportation coordination for maternal emergencies
Sunguya et al. (50)	Qualitative empirical study	Community/governance systems	Examines governance and sustainability lessons from emergency transportation scale-up
Kapologwe et al. (51)	Impact evaluation study	Community emergency systems	Demonstrates effectiveness of digitally coordinated emergency transport in improving maternal outcomes
Scott & Mars (52)	Conceptual review	Global digital health systems	Discusses telehealth implementation challenges and workforce readiness in low-resource settings
WHO (53)	Global digital health strategy	Global governance	Provides strategic direction for safe, equitable, and integrated digital health transformation
Ellaway et al. (54)	Conceptual/professionalism literature	Global workforce/professionalism	Discusses digital professionalism and social-media-related responsibilities in health professions
Gilson (55)	Conceptual health systems analysis	Global health systems/trust	Explores trust as a foundational component of healthcare systems and legitimacy
Horumpende et al. (56)	Empirical study	Community/pharmacy services	Examines antibiotic dispensing practices affecting patient safety and stewardship
Chipwaza et al. (57)	Empirical study	Community/patient behaviour	Investigates antimicrobial self-medication practices in Tanzania
Sangeda et al. (58)	Empirical study	Facility stewardship systems	Assesses implementation of antimicrobial stewardship programmes in Tanzanian hospitals
Mboya et al. (59)	Systematic review	Community/national AMR context	Synthesizes evidence on irrational antibiotic use and AMR risks

The conclusive evidence base comprised:

- Twenty-two empirical quantitative, qualitative, and mixed methods studies.
- Ten policy, strategy, and regulatory documents.
- Eight programme and implementation reports.
- Seven global guidance and normative documents; and
- Six systematic reviews, conceptual reviews, commentaries, and implementation frameworks.

Given the heterogeneous nature of the evidence base, which included empirical research, policy documents, programme reports, implementation evaluations, and conceptual literature, no formal quality appraisal, risk-of-bias assessment, or evidence-grading process was undertaken. Instead, source credibility was assessed through systematic source charting, categorization by evidence type and provenance, and cautious interpretation of findings according to their methodological strength, contextual relevance, and consistency across sources. Greater interpretive weight was assigned to empirical studies, systematic reviews, national policy documents, and authoritative guidance documents, while programme reports and conceptual papers were primarily used to provide contextual and implementation insights that complemented empirical findings.

### 3.8 Synthesis process

Thematic synthesis was conducted using an iterative interpretive approach supported by NVivo 12 software (Lumivero, formerly QSR International). Initial coding focused on recurring patterns related to implementation experiences, governance challenges, workforce preparedness, patient behaviours, service-delivery transformation, and safety concerns. Codes were progressively refined and consolidated into six interconnected thematic domains: (i) digital health governance and interoperability; (ii) facility systems and workflow transformation; (iii) data quality, documentation practices, and data use; (iv) virtual care models and digitally enabled service delivery; (v) artificial intelligence-enabled diagnostics and emerging workforce demands; and (vi) the rise of e-patients and informal or insufficiently regulated digital care pathways.

The thematic synthesis was also informed by a practice-based perspective. In addition to identifying policy and implementation themes, the analysis examined how digital health work is enacted through situated practices involving healthcare workers, patients, informal e-providers, tools and technologies, communication routines, documentation practices, referral processes, privacy behaviours, and stewardship responsibilities. This lens helped identify mismatches between formal digital health procedures and actual practices emerging in digitally mediated care environments.

To strengthen analytical rigor, findings were interpreted according to their evidentiary context. Distinctions were maintained between Tanzania-specific empirical evidence, evidence derived from broader sub-Saharan African settings, and insights drawn from global digital health, implementation science, and virtual care literature. This approach minimized overgeneralization and ensured that conclusions reflected the relative strength and geographical relevance of available evidence, particularly in areas where Tanzania-specific data remain limited.

The synthesis also informed the development of the TRUST framework. Through iterative comparison of findings across source categories, recurrent themes relating to provider verification, clinical governance, accountability, confidentiality, data protection, medication safety, antimicrobial stewardship, and evolving patient–provider relationships were mapped into framework domains. This process enabled the integration of empirical evidence, implementation experience, and policy considerations into a contextually grounded framework intended to support workforce preparedness, regulatory readiness, and accountable digital health implementation in Tanzania and comparable low- and middle-income country settings.

## 4 Results

### 4.1 Theme 1: digital health governance and interoperability

*Key message: Tanzania is shifting from “many digital projects” to “one digital ecosystem*”*.*

This theme is supported primarily by Tanzania-specific national policy documents, interoperability frameworks, investment roadmaps, and implementation reports that directly describe governance reforms, interoperability initiatives, and digital health coordination mechanisms in Tanzania (3–7). Additional support is drawn from WHO global digital health strategies and implementation guidance emphasizing governance, integration, and equitable digital transformation (1, 2). In general, the evidence for governance reform and interoperability efforts is relatively strong at policy and programme level. However, direct empirical evidence linking these reforms to patient trust, workforce behaviour, and long-term service legitimacy remains limited and partly inferential. The specific evidence is presented next.

Tanzania's national digital health strategies and governance documents consistently describe a transition from fragmented, project-based digital initiatives toward a coordinated and interoperable digital health ecosystem. An example is the National Digital Health Strategy (2019–2024) that explicitly acknowledges the challenge of multiple digital systems operating in parallel with limited coordination and interoperability and therefore prioritizes governance, interoperability, standards-based information exchange, and safe sharing of health information (4). Similarly, the Tanzania Digital Health Investment Road Map (2017–2023) frames investments in digital systems, data use, and workforce strengthening as critical mechanisms for improving service delivery, decision support, accountability, and continuity of care across the health system (6). These priorities are also aligned with Tanzania's broader eHealth direction established through earlier national strategies emphasizing ICT-enabled health service improvement and workforce preparedness (3).

Policy documents further indicate that a major operational step toward integration has been Tanzania's investment in interoperability and health information exchange mechanisms. A detailed national implementation report describing the country's interoperability journey between 2014 and 2019 documented the use of governance structures, enterprise architecture, standards, programme management, capacity building, and data-use approaches to connect previously fragmented systems through a national interoperability layer (7). The report further noted improvements in data availability, information exchange, and operational efficiency, while also highlighting the transition of system ownership and management to Tanzanian institutions. Complementing these efforts, the Tanzania Health Enterprise Architecture document provides a broader governance blueprint designed to align digital solutions with national health-sector priorities, reduce duplication, strengthen accountability, and improve coordination across systems and programmes (5).These governance reforms appear to be consistent with broader WHO digital health strategies and guidance emphasizing that digital transformation should be integrated, equitable, interoperable, and supported by strong governance mechanisms rather than implemented as isolated technological projects (1, 2, 14). Collectively, the evidence suggests that Tanzania is no longer debating whether digital transformation is necessary; rather, the central challenge is how to implement digital health in ways that strengthen accountability, legitimacy, workforce readiness, continuity of care, and public trust.

### 4.2 Theme 2: healthcare facility systems and workflow

*Key message: Healthcare facility digitalization is improving visibility and efficiency, but training and usability gaps remain among healthcare workers*.

This theme is supported by Tanzania-specific empirical studies, operational manuals, implementation reports, and user-experience evaluations examining GoTHOMIS implementation, eHealth competencies, workflow adaptation, and healthcare workers' experiences with digital systems in facility settings (29–36). WHO recommendations on digital interventions and telemedicine implementation further reinforce the importance of workforce preparedness and workflow integration in digital health systems (1, 2, 39). Evidence directly demonstrates documentation burden, workflow inefficiencies, infrastructure limitations, and training gaps associated with digital system implementation (31–36). However, broader implications for patient trust, digital professionalism, and long-term organizational adaptation are interpreted using implementation science and workforce literature rather than directly measured outcomes (33–38).

Evidence suggests that, at the healthcare facility level, Tanzania has prioritized hospital information systems as a foundation for operational digitalization and service-delivery coordination. The GoTHOMIS is presented within government programme documentation as a national health management information system designed to strengthen healthcare facility operations, improve reporting, support continuity of care, and enhance management of service delivery across levels of the health system (29, 30). Operational manuals and end-user training materials further describe GoTHOMIS as integrating electronic medical records, patient registration, outpatient and inpatient workflows, laboratory and pharmacy management, patient wallet functions, reporting systems, and inventory management into a unified digital platform intended to improve efficiency and accountability within healthcare facilities (30). These reforms align with Tanzania's broader digital health governance agenda emphasizing interoperability, coordinated data systems, and integrated service delivery (3–7).

User-experience evidence suggests that healthcare workers generally recognize the potential value of digital systems while continuing to face substantial day-to-day implementation constraints. A Tanzanian evaluation comparing systems such as GoTHOMIS, Care2x, and AfyaPro reported perceived benefits related to information visibility, record management, and workflow coordination, while simultaneously documenting persistent challenges associated with unstable infrastructure, unreliable internet connectivity, limited technical support, and continuing training needs among healthcare workers (31). Similar findings emerge from broader digital health implementation literature in low-resource settings, where technology adoption is strongly shaped not only by system functionality but also by contextual issues such as organizational support, usability, infrastructure reliability, and perceived usefulness (33–38, 40–42).

Workforce competence studies further reinforce the importance of human capacity development in successful digital transformation. A mixed-methods study conducted in private health centres in urban Tanzania found substantial variation in healthcare workers' computer literacy, informatics competencies, and eHealth preparedness, with limited exposure and insufficient training constraining confidence and effective system use despite generally positive attitudes toward digital health technologies (32). These findings are consistent with WHO digital health guidance emphasizing that workforce readiness is central to safe and sustainable digital health implementation (1, 2, 39). In addition, implementation science frameworks such as NASSS and PRISM suggest that successful scale-up of digital systems depends on continuous adaptation between technologies, users, organizational contexts, and health-system structures rather than technology deployment alone (17, 19).

Recent evidence examining HMIS workflows and electronic-record usability in Tanzanian healthcare settings further highlights the importance of workflow integration and user-centered implementation approaches (25–36). Studies assessing hybrid paper–digital systems have shown that parallel reporting processes, unreliable infrastructure, and inconsistent integration between systems may increase workload, reduce efficiency, and create frustration among healthcare workers (34–36). Qualitative evidence additionally suggests that healthcare workers often perceive digital systems positively when they improve information retrieval and continuity of care, but negatively when they duplicate tasks or disrupt routine clinical workflows (36). These operational challenges are important because they may indirectly affect patient perceptions of competence, continuity, and trust in digital care environments, even though such trust outcomes have not been directly measured in most Tanzanian studies.

Overall, the available evidence indicates that digital health scale-up in healthcare facilities is not simply a software or hardware issue but also a workforce development, workflow adaptation, and organizational governance challenge. Sustained success therefore depends on continuous healthcare worker training, supportive supervision, infrastructure reliability, usability-oriented system design, and alignment between digital technologies and real clinical workflows.

### 4.3 Theme 3: data quality and use

*Key message: Routine data systems show persistent quality and usage challenges in hybrid environments*.

This theme is grounded primarily in Tanzania-specific empirical studies examining HMIS data quality, data utilization, hybrid paper–digital workflows, and workforce challenges affecting documentation practices and continuity of care (34–36). National digital health strategies and interoperability frameworks further support the importance of integrated information systems and dependable data governance for accountability and service delivery improvement (4–7). The evidence directly demonstrates challenges related to data accuracy, workflow duplication, infrastructure reliability, and documentation practices within healthcare facilities. However, conclusions regarding how these operational inefficiencies influence patient perceptions of competence, continuity, and trust are interpretive and informed additionally by broader trust and implementation literature (29–32, 38, 39, 43–45) rather than directly demonstrated in Tanzania-specific studies.

Literature indicates that the Tanzania's digital health ecosystem includes national routine reporting systems, district-level reporting platforms, and hybrid workflows in which paper-based documentation and digital systems frequently coexist. Evidence suggests that maintaining data quality and promoting routine data use remain major implementation challenges despite substantial progress in digitalization efforts. A national assessment examining routine HMIS data quality in primary healthcare facilities and district systems found weaknesses in data accuracy, consistency, and completeness, with information reported into DHIS2 not always corresponding to underlying paper-based facility registers (34). The study highlighted that healthcare workers often operate within complex documentation environments requiring parallel entry into multiple systems, increasing the risk of discrepancies, delayed reporting, and documentation fatigue. The authors further emphasized that improving data quality requires targeted capacity building, supportive supervision, and stronger data-management competencies among healthcare workers at both facility and district levels.

Related evidence examining HMIS data utilization also suggests that the presence of digital systems alone does not guarantee meaningful use of information for planning and decision-making. A national study assessing HMIS data utilization found that although many healthcare workers reported using routine HMIS data in their daily work, regular data analysis and evidence-informed decision-making at facility and district levels remained inconsistent (35). The study further identified several implementation barriers, including inadequate resources, limited technical support, insufficient supervision, weak standard operating procedures, and gaps in recent HMIS-related training among healthcare workers. More recent qualitative evidence provides additional insight into the practical realities healthcare workers face within hybrid documentation environments. A qualitative case study examining perinatal data systems in Kilimanjaro described how simultaneous use of paper-based and digital systems contributed to inefficiencies related to manual data entry, duplication of tasks, retrieval challenges, storage limitations, and interruptions in information flow (36). Healthcare workers participating in the study generally expressed positive attitudes toward the long-term potential of fully digital systems; however, they emphasized that successful implementation would require reliable infrastructure, including stable electricity and internet connectivity, integrated systems that minimize redundancy, and ongoing practical training and technical support. Similar concerns regarding workflow burden, documentation duplication, and infrastructure instability have also been reported in broader facility-level digital health implementation evaluations in Tanzania (29–32).

These findings align with broader implementation science perspectives, which emphasize that successful digital transformation depends not only on the availability of technology, but also on organizational readiness, workforce competence, workflow integration, and continuous institutional support (29–32, 38, 39, 43–45). Similarly, technology acceptance and sustainability frameworks suggest that healthcare workers are more likely to engage meaningfully with digital systems when such technologies are perceived as usable, supportive of routine clinical workflows, and relevant to day-to-day service delivery needs (17, 19, 20). Consequently, efforts to improve data quality and data use should extend beyond strengthening technology adoption to include sustained investments in workforce preparedness and supportive implementation environments.

These operational realities are important because routine patient experiences are shaped not only by clinical outcomes but also by how efficiently healthcare systems function during care delivery. Delays associated with system downtime, repeated questioning resulting from fragmented records, missing information, or inconsistent documentation may indirectly affect patient confidence in healthcare workers and digital systems, even where clinical care itself remains appropriate. Although Tanzanian studies have not directly measured the relationship between data-quality challenges and patient trust, broader literature on healthcare legitimacy and trust suggests that patients frequently interpret inefficiencies, poor coordination, and repeated administrative failures as indicators of weak organizational competence and accountability (39, 42–45).

Overall, the evidence suggests that digitalization without sufficient workflow redesign, infrastructure reliability, workforce training, and organizational support may unintentionally increase workload, frustration, and operational inefficiencies rather than strengthen continuity, accountability, and trust in healthcare delivery.

### 4.4 Theme 4: AI-enabled diagnostics

*Key messages: AI-enabled digital diagnostics are expanding, increasing interpretive demands on the healthcare workforce*.

This theme is supported by Tanzania-specific diagnostic validation studies, telepathology implementation research, and emerging AI-supported respiratory diagnostic initiatives (37–41). WHO ethical and governance guidance for AI in health provides additional global evidence supporting the need for transparency, accountability, workforce preparedness, and patient protection in AI-supported healthcare environments (39). The available evidence demonstrates growing implementation of AI-supported diagnostics and highlights emerging workforce interpretive demands related to algorithm-supported decision-making (37–41). However, evidence regarding long-term workforce adaptation, patient trust in AI-assisted decisions, safety outcomes, and clinical integration remains relatively limited and partly anticipatory, requiring cautious interpretation informed by broader digital health implementation frameworks.

Documents suggest that Tanzania's digital transformation is increasingly extending beyond administrative and reporting functions toward clinical diagnostics, decision support, and digitally assisted interpretation. An example is the tuberculosis diagnosis where computer-aided detection technologies such as CAD4TB have been evaluated within Tanzanian populations as part of broader efforts to strengthen diagnostic capacity in resource-limited settings. A validation study examining CAD4TB performance reported that the system was able to distinguish chest radiographs of culture-positive tuberculosis cases from controls and suggested that further operational, ethical, and cost-effectiveness evaluations were needed before wider integration into diagnostic algorithms (37). These findings indicate that AI-supported diagnostics may improve access to interpretation support in settings facing radiological workforce shortages, while also introducing new governance and training demands related to interpretation of algorithmic outputs and management of diagnostic uncertainty.

Existing evidence shows that Tanzania is also contributing to emerging non-invasive AI-supported diagnostics through respiratory cough-audio research. A recently published diagnostic protocol described development and evaluation of an AI-based cough classifier intended to support detection of respiratory conditions such as tuberculosis, asthma, and chronic obstructive pulmonary disease within rural Tanzanian contexts (40). The related “Kikohozi classifier” initiative, described within UKRI programme documentation, is currently being implemented across multiple regions of Tanzania with the goal of improving respiratory disease diagnosis and treatment monitoring using AI-powered cough analysis adapted for low-resource environments (41). These initiatives are particularly relevant because they illustrate how AI-supported diagnostics are increasingly being designed for frontline and community-level use rather than remaining confined to highly specialized facilities.

It is important to note that as these technologies expand, healthcare workforce roles are also changing. Clinicians, nurses, laboratory personnel, and allied healthcare workers increasingly require competencies beyond traditional clinical interpretation. They must be able to interpret algorithmic outputs, recognize uncertainty, communicate risks effectively, and document decisions transparently. WHO guidance on ethics and governance of AI for health specifically emphasizes transparency, accountability, explainability, and preservation of public trust as essential principles when AI systems influence clinical decision-making (38). These emerging interpretive demands are also consistent with broader literature on digital professionalism, virtual care governance, and technology adoption, which highlights that workforce confidence and patient trust are strongly shaped by how healthcare workers integrate digital tools into routine clinical interactions (46–52).

At the same time, the evidence demonstrates that AI-supported diagnostics should not be interpreted as replacing healthcare workers but rather as reshaping clinical decision-support environments. Current Tanzanian evidence primarily demonstrates technical feasibility, pilot implementation, and diagnostic potential rather than long-term effects on clinical outcomes, workforce behaviour, or patient trust (39, 40). Consequently, several conclusions regarding future workforce transformation, patient acceptance of AI-supported care, and broader implications for clinical legitimacy remain interpretive and should be approached cautiously pending additional empirical evaluation within routine healthcare settings.

### 4.5 Theme 5: virtual healthcare care transformations

*Key message: Virtual care is growing, but sustainability depends on governance, financing and training*.

This theme is supported by Tanzania-specific teleconsultation studies, programme evaluations, qualitative community-based research, and digitally enabled referral and emergency transportation initiatives (33, 44–51). Regional and global telemedicine literature additionally provides compelling evidence regarding governance, workforce, infrastructure, financing, and implementation challenges affecting sustainability of virtual care systems (34, 35, 44). Empirical evidence from Tanzania strongly supports the feasibility and effectiveness of digitally coordinated referral systems, tele-support models, and emergency transportation innovations (46–51). Nevertheless, conclusions regarding broader virtual care legitimacy, regulatory readiness, workforce transformation, and patient trust remain partly inferential because longitudinal evidence on routine virtual care integration in Tanzania remains limited.

Evidence suggests that virtual care in Tanzania increasingly includes patient-facing teleconsultations, provider-to-provider communication and mentorship, digitally supported referral coordination, and emergency transportation systems. At policy and implementation levels, Tanzania-focused reviews consistently identify recurring barriers affecting telemedicine implementation, particularly in rural and underserved settings. These barriers include unstable telecommunications infrastructure, unreliable internet connectivity, inadequate healthcare workers' digital literacy, financing constraints, and insufficiently developed regulatory and governance frameworks for virtual care delivery (33). Similar implementation challenges have been documented across sub-Saharan Africa, where broader systematic reviews identify workforce limitations, infrastructure gaps, financing constraints, and weak policy coordination as major barriers to sustainable telemedicine scale-up (42). WHO implementation guidance aligns closely with these observations by emphasizing that successful telemedicine implementation requires structured situational assessment, governance mechanisms, implementation planning, workforce preparation, monitoring systems, and continuous evaluation rather than informal or isolated digital initiatives (43).

Tanzania's COVID-19 experience provides an important real-world example of both increased demand for virtual care and the practical constraints affecting sustainability. An audit examining teleconsultation trends at a tertiary healthcare facility during the COVID-19 pandemic documented increased use of teleconsultation services while simultaneously identifying financing mechanisms, reimbursement systems, and insurance coverage limitations as significant barriers to long-term sustainability (44). These findings suggest that virtual care expansion depends not only on technological availability but also on broader organizational and financial systems capable of supporting routine service delivery. Similarly, telepathology implementation studies from Northern Tanzania demonstrate how digital technologies are increasingly supporting specialist diagnostic services across geographical distances. An implementation study examining telepathology for cervical lesion diagnosis showed that scanned digital images could successfully support diagnostic interpretation and specialist consultation; however, sustainability depended heavily on practical issues such as hardware functionality, internet connectivity, technical support, and workflow integration within healthcare facilities (38).

In addition, evidence from rural chronic disease support interventions demonstrates that trust and legitimacy remain central to successful uptake of digitally mediated healthcare communication. A qualitative study of mobile support for noncommunicable diseases in rural Tanzania found that uptake depended heavily on trust in healthcare workers and the perceived legitimacy of the information source (45). Existing community relationships also influenced acceptance. Community health workers participating in the study also emphasized the need for training, supervision, and institutional support when assuming expanded digital communication responsibilities. These findings are consistent with broader trust and digital professionalism literature suggesting that patient acceptance of virtual care is strongly influenced by confidence in healthcare workers and perceived reliability of digital interactions (1, 2, 52–55). Studies on provider-to-provider virtual collaboration models have also demonstrated important potential for workforce strengthening and quality improvement. A mixed-methods assessment of a multimodal surgical mentorship intervention implemented in Tanzania's Lake Zone reported that telementoring, WhatsApp-supported consultation, and virtual case discussion, when integrated with in-person mentorship and supervision, could strengthen teamwork, clinical confidence, and quality improvement within healthcare facilities (46). The study further suggests that digital communication tools are most effective when embedded within supportive governance structures and ongoing professional mentorship rather than functioning as isolated technological interventions. These findings align with broader implementation and workforce-development literature emphasizing that successful digital transformation depends on continuous workforce support, organizational adaptation, and supervision systems (16–20).

Most importantly, documents on several large Tanzanian programmes illustrate that virtual care increasingly functions as digitally coordinated care pathways rather than teleconsultation alone. The Afya-Tek programme, for example, was designed to strengthen continuity of care by digitally linking community health workers, healthcare facilities, and accredited drug dispensing outlets through referral systems and digital decision-support tools integrated within government health systems (47). Similarly, the m-mama programme demonstrates how digital technologies can support remote triage, ambulance dispatch, and activation of community transportation systems for maternal and newborn emergencies in resource-limited settings. Peer-reviewed evaluations of these programmes describe important lessons regarding governance, sustainability, scalability, coordination, and cost-effectiveness within Tanzanian contexts (48–51). These interventions illustrate that virtual care transformation in Tanzania increasingly involves digitally enabled coordination across multiple levels of the health system, including community-based care, referral networks, and emergency response mechanisms.

Overall, current evidence indicates that sustainability of virtual care depends not only on technological innovation and workforce competence but also on governance structures, financing arrangements, supervision systems, infrastructure reliability, reimbursement mechanisms, and public trust. Although Tanzania has demonstrated substantial innovation in digitally coordinated care models, stronger longitudinal evidence is still needed regarding routine integration of virtual care into health systems, workforce adaptation, patient safety, regulatory oversight, and long-term trust in virtual care settings.

### 4.6 Theme 6: the rise of e-patients and e-providers

*Key message: e-patients and informal e-providers are emerging as influential in pluralistic medical systems*.

Evidence supporting this theme remains comparatively limited and is derived from a combination of Tanzania-specific conceptual analysis, global literature on Medicine 2.0, online health-information seeking, eHealth literacy, digital professionalism, and comparable evidence on digital patient behaviour and trust in healthcare interactions (8–12, 45). Tanzania-specific evidence directly examining the emergence of e-patients and informal digital consultations remains limited largely to narrative, conceptual, and interpretive analyses rather than large empirical studies (11). Broader evidence from systematic reviews and conceptual literature nevertheless supports concerns regarding online misinformation, changing therapeutic relationships, digital professionalism, trust in healthcare workers, and the growing influence of digitally mediated healthcare interactions on patient decision-making (8–10). Existing implementation and trust literature further suggests that patient engagement with digital health technologies is strongly shaped by perceived legitimacy, accessibility, usability, and confidence in both providers and digital platforms (33–38, 45). Consequently, several conclusions regarding the scale, characteristics, and health-system consequences of informal digital consultations and non-credentialed e-providers in Tanzania should be interpreted as contextually grounded and theoretically informed inferences rather than directly demonstrated causal relationships.

The available literature suggests that as digital health ecosystems expand, patient behaviour changes in predictable ways. Increasing numbers of people now search for medical information online, compare health advice across platforms, participate in digital health communities, and seek consultation from both formal and informal digital sources before or after engaging with healthcare facilities. Medicine 2.0 literature describes a shift from passive receipt of information to participatory and digitally mediated health engagement. Patients increasingly act as information seekers, interpreters, and contributors within online environments (8, 9). Tanzania-focused scholarship similarly describes a growing ecosystem of informal or insufficiently regulated digital consultations accessed through social media platforms and mobile applications, where informal e-providers may offer health advice, recommendations, or medicines outside established systems of professional accountability and regulatory oversight (11).

This transformation appears to reflect both demand-side and supply-side pressures within healthcare systems. On the demand side, patients may perceive informal digital channels as faster, cheaper, more convenient, less stigmatizing, or more responsive than formal healthcare services, particularly where health facilities are overcrowded, geographically inaccessible, or associated with long waiting times. On the supply side, rapid growth in mobile-phone use, social media engagement, and digital communication platforms has expanded opportunities for informal health-related communication and advice-sharing outside traditional clinical settings (8, 9). Broader literature on online health-information seeking further demonstrates that patients increasingly compare advice from multiple sources, including healthcare workers, online forums, social media, and peer networks before making healthcare decisions (10, 12). Importantly, evidence also suggests that the quality of the patient–provider relationship strongly influences whether online information contributes to constructive engagement and self-care or instead amplifies confusion, misinformation, anxiety, and mistrust (10).

Qualitative evidence from Tanzania additionally highlights the importance of trust, legitimacy, and perceived credibility within digital healthcare interactions. A rural Tanzanian study examining mobile approaches for chronic disease support found that uptake of digital communication depended heavily on trust in the sender, community relationships, and perceived legitimacy of the healthcare worker or information source (45). These findings are consistent with broader literature on digital professionalism and healthcare legitimacy, which emphasizes that patient trust is not determined only by technical accuracy but also by how healthcare workers communicate, demonstrate professionalism, and maintain accountability in digital spaces (54, 55). Emerging evidence from sub-Saharan Africa similarly suggests that uptake and sustained use of digital and virtual care services are strongly influenced by perceived provider legitimacy, responsiveness, interpersonal communication, and trust in healthcare workers, particularly in settings where formal and informal health information sources coexist and compete for public attention (42, 45, 52). In digitally mediated environments where formal and informal sources compete for attention, patients may therefore judge credibility not only on professional qualifications but also on accessibility, responsiveness, communication style, and perceived empathy.

The practical implication is that e-patients themselves should not be viewed as a problem or a deviation from formal healthcare systems. Instead, they represent a predictable feature of contemporary digital health environments in which patients increasingly participate actively in information-seeking and healthcare decision-making (8–12, 45, 54, 55). The greater risk arises when formal healthcare systems fail to engage constructively with digitally informed patients, leaving online health spaces dominated by misinformation, unverified providers, and commercially driven health advice. In such environments, absence of visible professional engagement and trusted regulatory oversight may contribute to erosion of confidence in formal healthcare workers and increase exposure to unsafe digital consultations, inappropriate prescribing, and misleading health information. Although direct empirical evidence quantifying these risks in Tanzania remains limited, the convergence of global evidence on online health behaviour, trust, and digital professionalism suggests that health systems must increasingly consider e-patient engagement and digital trust-building as central components of modern healthcare governance rather than peripheral concerns.

## 5 Discussion

Viewed through a practice lens, the findings suggest that Tanzania's digital health transition is fundamentally a transformation of healthcare practices rather than a purely technological or policy implementation process. Practice scholars argue that organizational change occurs through situated practices including the recurrent activities through which people work, interact, make decisions, use technologies, and respond to contextual demands (21–23). From this perspective, digital health is enacted through everyday practices involving healthcare workers, patients, digital technologies, data systems, and governance arrangements. Digital health is therefore not simply a collection of technologies or policies but is enacted through everyday practices, particularly how healthcare workers communicate, document, prescribe, refer, supervise, interpret digital outputs, protect confidentiality, and engage with digitally informed patients; how patients seek advice, compare information, evaluate provider credibility, and navigate formal and informal sources of care; and how digital technologies shape the organization and delivery of healthcare (25, 28). Consequently, the success of digital transformation depends not only on the availability of policies, platforms, and infrastructure, but also on how these are incorporated into routine clinical work, patient interactions, documentation practices, referral processes, prescribing decisions, privacy protection, and accountability mechanisms (25, 28).

A first major insight is that Tanzania has established substantial policy and governance foundations for digital transformation, including national digital health strategies, interoperability frameworks, enterprise architecture approaches, and digital investment roadmaps (4–7). These reforms align closely with WHO recommendations emphasizing integrated, interoperable, and workforce-supported digital transformation (1, 2, 39). However, evidence from healthcare facilities and district-level implementation studies suggests that the practical enactment of these policies remains uneven. Challenges related to digital competencies, infrastructure instability, workflow duplication, documentation burden, and inconsistent use of routine data continue to shape how digital systems function in everyday care settings (29–36). These findings highlight a recurring implementation gap between formal digital health architecture and the realities of frontline practice. Importantly, patients increasingly experience digital systems directly through registration processes, documentation workflows, referral systems, and clinical encounters. As a result, the quality of digital health practices not merely the existence of digital systems, can influence perceptions of competence, continuity of care, and trust.

A second major insight is that digital transformation is reshaping healthcare work itself. Virtual communication, digital documentation, AI-assisted diagnostics, teleconsultations, and digitally enabled referral systems are creating new expectations and responsibilities for healthcare workers (8–12, 37–41). In practice, healthcare workers are increasingly required to combine clinical expertise with competencies in digital communication, patient verification, privacy protection, interpretation of technology-supported outputs, and engagement with digitally informed patients. Evidence from Tanzania and comparable settings demonstrates that digital technologies can strengthen coordination, mentorship, referral systems, and diagnostic support while simultaneously generating new demands related to workflow adaptation, communication, accountability, and clinical judgment (32, 37–51). These findings suggest that workforce preparedness should be viewed not simply as technical training but as preparation for new forms of digital health practice.

A third major insight is the growing interaction between Tanzania's formal digital health ecosystem and an expanding informal digital health environment. The literature suggests that e-patients are becoming increasingly common as individuals seek health information online, compare advice across multiple platforms, and engage with both formal and informal digital sources of care (8–12). Tanzania-focused analyses further point to the emergence of informal digital consultations occurring through social media platforms, messaging applications, and other digital channels that may operate outside established accountability and regulatory mechanisms (11). From a practice perspective, these developments represent the emergence of new health-seeking and care-delivery practices rather than simply alternative information sources. Although the scale and consequences of these practices remain insufficiently documented, they are likely to influence how patients assess provider credibility, make healthcare decisions, and engage with formal health services. The challenge for health systems is therefore not merely to regulate informal digital spaces but to understand and respond to the realities of patient behaviour within increasingly pluralistic digital health environments.

A fourth major insight concerns patient safety and stewardship within evolving virtual care practices. Existing evidence demonstrates persistent challenges related to inappropriate antimicrobial use, self-medication, and inconsistent implementation of stewardship programmes in Tanzania (14, 15, 56–59). As virtual consultations, e-prescribing, online medicine access, and digitally mediated advice become more common, these practices may introduce new risks if adequate supervision, documentation, referral mechanisms, and accountability structures are not established. Although direct evidence regarding digital prescribing and online medicine access remains limited, the findings suggest that stewardship should be understood as a broader practice-governance issue encompassing supervision of virtual care, safe prescribing behaviours, referral decisions, patient follow-up, and monitoring of digitally mediated clinical interactions.

Taken together, these findings suggest that Tanzania's digital health transition is increasingly a question of how digital health work is practiced, governed, and trusted in everyday healthcare environments. The evidence points to an emerging gap between formal digital health policies and the realities of e-patient, e-provider, e-consultation, and digitally mediated care practices already occurring across the health system. This observation provides the rationale for the TRUST framework proposed in this manuscript. The existing implementation science frameworks provide valuable insights into adoption, implementation, sustainability, and technology acceptance (16–20). However, these frameworks pay comparatively limited attention to the situated digital health work practices through which healthcare workers, patients, technologies, and governance mechanisms interact in everyday care environments (21, 23, 26). The findings therefore suggest a need for a complementary practice-based framework capable of addressing the workforce, governance, relational, stewardship, technological, and accountability dimensions of digital health work. The TRUST framework responds to this need by focusing on the everyday practices through which trust, legitimacy, safety, confidentiality, accountability, and quality are established and maintained in environments where formal and informal digital care increasingly coexist. In this way, TRUST serves as a bridge between digital health policy aspirations and the practical realities of e-patient, e-provider, virtual care, and digitally mediated healthcare practices in Tanzania and similar settings.

## 6 The TRUST framework: a practice-based perspective for trustworthy digital health and virtual care

The evidence synthesized across the six themes consistently highlighted challenges surrounding provider legitimacy, governance, patient engagement, safety, and trust. This points to the need for a practical framework capable of connecting digital health governance, workforce preparedness, patient trust, confidentiality, stewardship, regulatory accountability, and technology-enabled care practices within rapidly evolving digital health environments. Existing implementation science and digital health frameworks provide important conceptual foundations for understanding technology adoption, implementation, scale-up, sustainability, and user acceptance. For example, CFIR provides a comprehensive structure for understanding multilevel determinants influencing implementation success across intervention, organizational, and contextual domains (16). The NASSS framework offers valuable insights into factors affecting non-adoption, abandonment, scale-up, spread, and sustainability of complex health technologies (17). RE-AIM and PRISM emphasize implementation reach, effectiveness, sustainability, contextual fit, and organizational adaptation within real-world healthcare systems (18, 19), while TAM helps explain technology adoption through perceived usefulness and ease of use (20). However, the evidence synthesized in this review suggests that these frameworks do not fully address the practical and governance challenges emerging within Tanzania's evolving digital health ecosystem. In particular, the reviewed literature highlights tensions occurring at the intersection of workforce behaviour, patient trust, provider legitimacy, informal digital consultations, confidentiality, stewardship, digital communication, and accountability within increasingly complex digitally mediated care environments. Existing implementation frameworks primarily focus on explaining technology adoption and implementation processes and were largely developed in contexts where care delivery occurs predominantly within formal healthcare systems. They do not explicitly address settings where formal digital infrastructures coexist with informal mobile-phone-mediated consultations, social-media-based health advice, e-patient behaviours, and other emerging digital practices that increasingly influence healthcare delivery in many low- and middle-income countries (11).

Consistent with the practice-based perspective underpinning this review, digital transformation is understood not simply as the introduction of new technologies but as the emergence of new healthcare practices involving interactions among healthcare workers, patients, technologies, data systems, regulations, and institutional routines (21, 23, 26). From this perspective, trustworthiness in digital health depends not only on governance structures and workforce competence but also on how technologies are selected, configured, used, interpreted, and integrated into everyday clinical practice.

**Figure 1:**
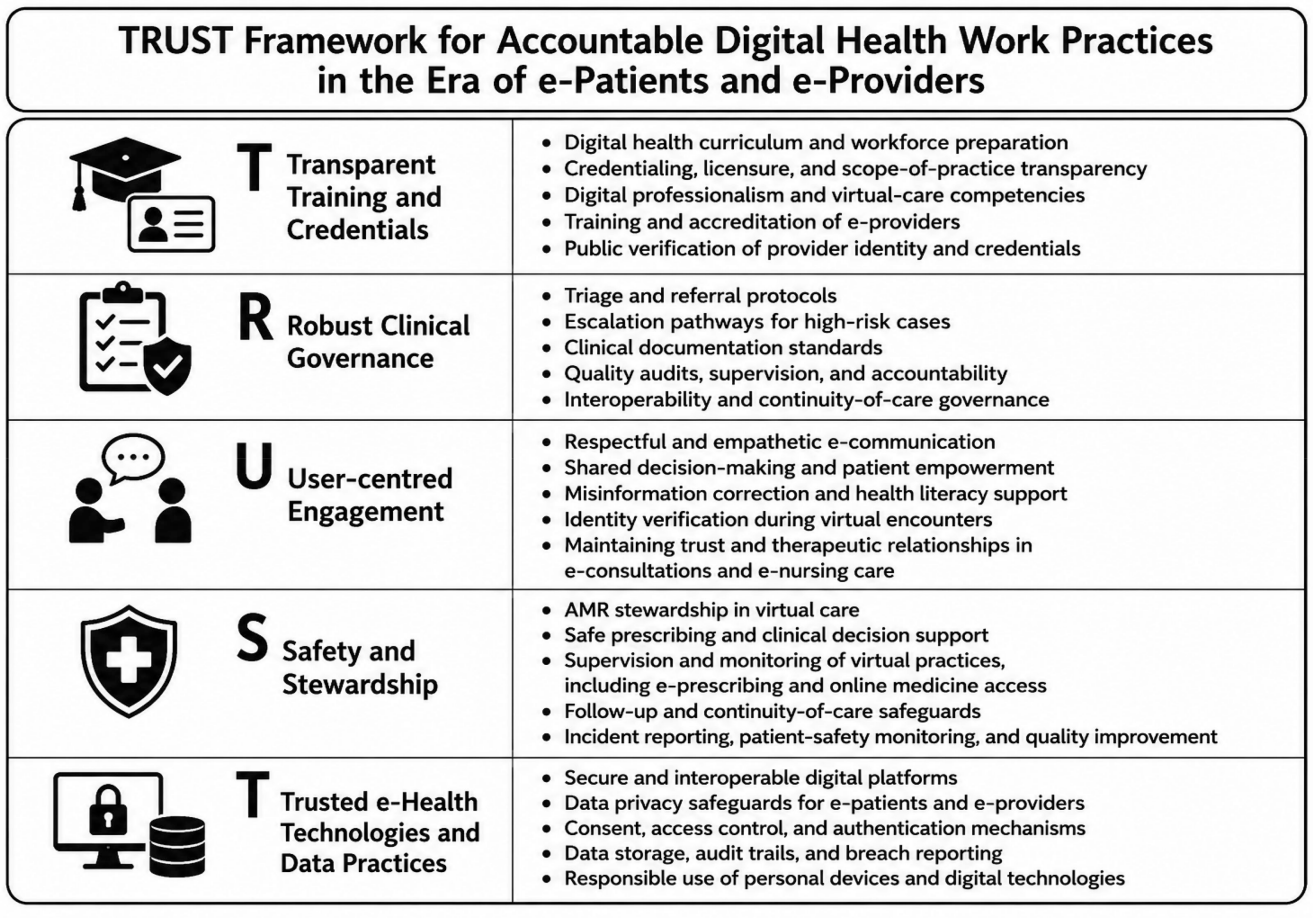
TRUST framework for accountable digital health work practices in the era of e-patients and e-providers.

The TRUST framework (Figure 1) was therefore developed abductively through iterative synthesis of findings from Tanzania-specific empirical evidence, implementation science literature, global digital health guidance, and practice-based scholarship. Across these sources, recurring concerns consistently emerged regarding provider verification, workforce readiness, digital communication, stewardship responsibilities, technological reliability, confidentiality, accountability, and legitimacy of digitally mediated care (Table 2). These concerns clustered into five interconnected practice domains: *Transparent credentials and workforce preparedness; Robust clinical governance; User-centred engagement; Safety and stewardship; and Trusted e-Health technologies and data practices*.

The framework is conceptualized as a practice-based operational framework rather than a technology-adoption model. It seeks to bridge formal policy intentions and the realities of frontline healthcare work by focusing on how healthcare workers, patients, technologies, and governance systems interact in everyday digital care environments. Future empirical validation involving patients, healthcare workers, regulators, professional councils, training institutions, and digital health implementers will be important for refining and contextualizing the framework across different healthcare settings. Although the five TRUST domains are presented separately for analytical clarity, they are highly interconnected in practice. Together, they address the workforce, governance, relational, safety, and technological dimensions that collectively shape trustworthiness in digital health and virtual care environments.

(i) **Transparent Training and Credentials for Digital Health Technologies and Virtual Care**

Virtual care requires systems that enable patients to verify provider identity, licensure status, and scope of practice as a foundation for trust and accountability. Evidence from Tanzania and broader digital health literature demonstrates that patients increasingly seek health information and consultations through digital channels, including informal and insufficiently regulated platforms, where provider credibility may be difficult to verify (8–11). At the same time, studies assessing healthcare workers' eHealth competencies in Tanzania have identified gaps in digital preparedness, informatics competence, and practical training needed for safe technology-supported care delivery (31–36). WHO digital health guidance similarly emphasizes that workforce readiness and governance are essential for safe digital transformation (1, 2, 39). Taken together, these findings suggest that trustworthy virtual care begins with confidence in both provider competence and provider legitimacy. Addressing these challenges therefore requires coordinated educational, regulatory, and credentialing responses.

These findings suggest the need for deliberate educational and regulatory responses from health training institutions, professional councils, and digital health governance structures. Strengthening digital competence should include telemedicine ethics, remote clinical decision-making, digital communication skills, patient verification protocols, confidentiality, and safe use of digital platforms in both pre-service and in-service training. Some of these competencies are already present within health professional curricula; however, the rapid expansion of virtual and informal digital care requires more explicit, standardized, and practice-based preparation. In addition, strengthening interoperable professional registries, digital credentialing systems, and publicly accessible verification mechanisms may help reduce impersonation, unsafe practice, and misinformation while improving public confidence in virtual care. Transparent credentialing therefore becomes both a workforce development strategy and a trust-building mechanism within digital health ecosystems. However, competent and identifiable providers alone are insufficient to ensure safe and accountable virtual care. These workforce capacities must be supported by governance structures that guide how digital care is delivered, monitored, and regulated.

(ii) **Robust Clinical Governance for Virtual Care**

Virtual care requires clear and enforceable governance structures for triage, documentation, escalation and referral pathways, supervision, audit, and continuous quality improvement. Evidence from Tanzania's digital health governance initiatives demonstrates increasing national emphasis on interoperability, coordinated digital architecture, and integrated data systems to improve accountability and continuity of care (4–7). At the same time, studies examining telemedicine implementation in Tanzania and sub-Saharan Africa consistently identify governance gaps, inconsistent regulation, infrastructure constraints, and workforce limitations as major barriers to safe and sustainable virtual care (42–49). These observations indicate that workforce preparedness must be complemented by organizational and regulatory systems capable of ensuring consistency, accountability, and quality across digital healthcare interactions.

Empirical evidence from healthcare facilities further shows that digital systems may increase workload, documentation burden, and workflow inefficiencies when governance structures, supervision, and training are insufficient (31, 32). Similarly, teleconsultation experiences during COVID-19 highlighted sustainability challenges related to financing, coordination, and operational oversight (44). These findings reinforce the importance of governance arrangements that define standards for remote prescribing, patient identification, continuity of care, digital documentation, and escalation of high-risk cases. Accountability mechanisms, including supportive supervision, digital audit trails, incident-reporting systems, and patient feedback mechanisms, are equally important for maintaining clinical quality and public trust.

Beyond operational oversight, governance also plays an important integrative role by ensuring that virtual care activities remain aligned with broader health-system goals and regulatory priorities. Importantly, governance structures for virtual care should not operate as parallel systems disconnected from national health priorities. Alignment with national digital health architecture, interoperability frameworks, and regulatory standards is necessary to ensure that digital services strengthen rather than fragment healthcare delivery (4–7). Robust clinical governance therefore functions as both a patient-safety mechanism and a legitimacy framework for accountable virtual care.

While governance structures create the conditions for safe and accountable care, the success of digital health ultimately depends on how patients and healthcare workers interact within these systems. This makes user engagement a critical component of trustworthy digital health practice.

(iii) **User-centered Engagement**

Digital transformation is reshaping the therapeutic relationship between patients and healthcare workers. Evidence from Medicine 2.0 and online health-information literature demonstrates that patients increasingly participate in digital networks, seek health information online, compare advice across multiple platforms, and arrive at healthcare encounters with pre-existing interpretations of illness and treatment options (8–10). Tanzania-specific analyses similarly suggest the emergence of e-patients who engage with informal digital consultations, social-media-mediated health advice, and other digitally enabled health-seeking behaviours outside traditional clinical settings (11). These developments highlight that digital transformation affects not only technologies and systems but also the social relationships through which healthcare is delivered and experienced.

From a practice-based perspective, these developments represent more than changes in information access; they reflect the emergence of new healthcare practices that alter how patients and providers communicate, negotiate decisions, establish trust, and share responsibility for care. Digital technologies create new opportunities for patient participation and continuity of care, but they also introduce new challenges related to legitimacy, misinformation, accountability, and maintenance of therapeutic relationships. These relational challenges become particularly visible in virtual care settings where traditional forms of clinical interaction are increasingly mediated through digital platforms.

Virtual care, including e-consultations and e-nursing care, reshapes the traditional clinical encounter by separating physical co-presence from communication, assessment, documentation, follow-up, and relational care. These interactions are therefore not simply conventional clinical encounters transferred to digital platforms. Rather, they constitute new situated practices in which trust, empathy, clinical judgment, communication, and accountability must be actively established and maintained through digital channels. Healthcare workers must often make clinical assessments with fewer sensory and contextual cues, while patients must place trust in providers whom they may never meet face-to-face. As a result, effective virtual care requires healthcare workers to develop new communication and relational competencies that extend beyond conventional clinical skills.

Within this context, healthcare workers require competencies that extend beyond technical proficiency. Effective digital engagement requires respectful, evidence-based, and non-defensive communication with digitally informed patients, together with the ability to explain uncertainty, support shared decision-making, and respond constructively to online health information and misinformation. Studies examining online health-information use demonstrate that the quality of the patient–provider relationship strongly influences whether digital information seeking enhances patient engagement or contributes to mistrust, confusion, and unsafe health behaviours (10). Evidence from community-based digital health interventions in Tanzania further highlights that trust, legitimacy, and respectful communication significantly influence uptake, sustained use, and perceived value of digital health services (45). These findings underscore that trust and engagement are not automatic outcomes of digitalization but must be actively cultivated through professional practice.

A practice-based perspective to user engagement therefore requires deliberate attention to the relational dimensions of virtual care. Healthcare workers should be equipped with competencies in empathetic e-communication, verification of patient and provider identity, explanation of the limits of remote assessment, privacy protection, informed consent, digital professionalism, and timely referral or escalation when physical examination or higher-level care is required. Equally important is the promotion of digital health literacy among patients so that they can critically evaluate health information, recognize credible providers, and participate safely in digitally mediated care.

User-centred engagement therefore extends beyond communication techniques alone. It encompasses the everyday practices through which patients and healthcare workers establish trust, negotiate decisions, exchange information, manage uncertainty, and maintain continuity of care within increasingly digital healthcare environments. When supported by appropriate governance, workforce competencies, and trustworthy technologies, these practices can strengthen access, patient empowerment, and continuity of care. Conversely, when provider identity, confidentiality, documentation, referral pathways, and follow-up mechanisms are unclear, digitally mediated interactions may undermine trust, patient safety, and accountability. User-centred engagement is therefore a critical practice domain for ensuring that digital transformation enhances rather than weakens therapeutic relationships and public confidence in healthcare systems. However, even strong patient-provider relationships cannot eliminate the clinical and organizational risks associated with digitally mediated care. Ensuring that virtual care practices remain safe therefore requires explicit attention to patient safety and stewardship.

(iv) **Safety and Stewardship**

From a practice lens, patient safety depends not only on technologies themselves but also on how digital and virtual care practices are performed, supervised, documented, and governed. As virtual care expands, healthcare increasingly occurs through e-consultations, e-prescribing, digital referral systems, telemonitoring, online medicine access, remote clinical decision-making, and informal digital advice exchanged through messaging platforms and social media. These emerging practices create important opportunities for improving access and continuity of care, but they also introduce new risks when professional standards, supervision mechanisms, documentation requirements, and accountability structures are unclear or inconsistently applied. Understanding these risks requires moving beyond a narrow focus on technology itself to consider how digital healthcare practices are governed and performed in everyday settings.

The evidence reviewed in this synthesis suggests that safety and stewardship concerns in digital health extend beyond medication use alone. Virtual care environments may create challenges related to provider verification, appropriateness of remote assessment, continuity of care, documentation quality, referral and escalation practices, follow-up arrangements, and accountability for clinical decisions. In settings where formal digital health systems coexist with informal digital consultations and social-media-mediated health advice, patients may encounter conflicting information, unclear provider responsibilities, or limited mechanisms for redress when adverse events occur. These risks underscore the importance of strengthening oversight and governance of virtual practices rather than focusing narrowly on individual technologies or online transactions. Among these broader safety concerns, medication-related risks remain particularly important because of their direct implications for patient outcomes and public health. Nevertheless, medication safety and antimicrobial stewardship remain critical components of safe virtual care. Tanzania continues to face substantial challenges related to inappropriate antimicrobial use, including non-prescription antibiotic dispensing, self-medication, and inconsistent implementation of antimicrobial stewardship programmes (14–15, 56–59). Expanding virtual consultations, e-prescribing, and online medicine access may create additional pathways for inappropriate prescribing, misinformation, delayed referral, and unsafe medicine use if safeguards are insufficient. National and global AMR strategies therefore remain highly relevant to the governance of virtual care systems (14–15). These challenges illustrate why stewardship should be viewed as an integral component of virtual care governance rather than a separate clinical activity.

The findings suggest that stewardship should be understood broadly as the responsible oversight of virtual care practices rather than solely as a medication-management function. Digital platforms and virtual care services should incorporate mechanisms for supervision and monitoring of e-consultations, e-prescribing, digital referral pathways, online medicine access, and other forms of digitally mediated care. Such mechanisms may include evidence-based prescribing guidance, clinical decision-support tools, referral and escalation protocols, documentation standards, audit systems, incident-reporting processes, and routine review of virtual care practices against professional and regulatory standards. Effective stewardship, however, depends not only on organizational systems but also on the behaviours and decisions of both providers and patients.

A practice-based perspective to safety and stewardship also requires attention to both e-patient and e-provider behaviours. Healthcare workers need competencies to recognize the limitations of remote assessment, communicate uncertainty appropriately, document virtual encounters adequately, and initiate timely referral when in-person evaluation is required. Patients similarly require support to recognize credible sources of advice, understand appropriate use of virtual services, and seek higher levels of care when necessary. Public digital-health communication can further contribute to patient safety by countering misinformation and promoting responsible use of medicines and healthcare services.

Safety and stewardship therefore function as mechanisms for ensuring that virtual care practices remain clinically appropriate, professionally accountable, and aligned with broader patient-safety and public-health goals. By strengthening supervision, monitoring, documentation, and accountability across digitally mediated care pathways, health systems can support innovation while reducing risks associated with poorly governed virtual practices. The effectiveness of these safety and stewardship mechanisms is linked to the trustworthiness of the technologies through which digital care is delivered. This highlights the importance of secure, reliable, and accountable digital platforms and data practices.

(v) **Trusted e-Health Technologies and Data Practices**.

Practice-based scholars emphasizes that technologies are not passive instruments but active components of healthcare practices that shape how work is performed, coordinated, documented, interpreted, and evaluated (23, 28). Consequently, trust in digital health depends not only on the behaviour of healthcare workers but also on the trustworthiness, usability, security, interoperability, and accountability of the technologies through which care is delivered. Technological trustworthiness therefore becomes a prerequisite for sustaining confidence in both digital health services and the institutions that provide them.

Evidence reviewed in this synthesis demonstrates that concerns regarding confidentiality, privacy, documentation quality, interoperability, and patient safety are linked to the design and use of digital technologies themselves. Tanzania's Personal Data Protection Act establishes important legal safeguards for protecting personal information (14), while national digital health architecture and interoperability initiatives seek to promote secure information exchange and accountable data governance (4–7). Nevertheless, implementation studies suggest that confidentiality risks may arise through insecure messaging platforms, shared devices, weak authentication systems, fragmented information systems, inadequate access controls, and inconsistent documentation practices (34–36, 46–51). Addressing these vulnerabilities requires a socio-technical perspective that recognizes the interdependence of technological safeguards and human practices.

A practice-based perspective suggests that trustworthy digital health requires attention to both technological and human dimensions. Digital platforms should therefore support secure authentication, provider verification, audit trails, interoperability, decision support, role-based access controls, breach-reporting mechanisms, and transparent governance arrangements. At the same time, healthcare workers require practical competencies related to digital documentation, secure communication, patient identification, informed consent, and responsible use of personal digital devices in clinical care.

Trusted e-Health technologies and data practices therefore extend beyond legal compliance or cybersecurity considerations alone. They encompass the broader socio-technical arrangements through which technologies, data, healthcare workers, patients, and governance systems interact to support safe, accountable, reliable, and trustworthy digital healthcare delivery. Strengthening these socio-technical practices is essential because patient willingness to engage with virtual care depends not only on confidence in providers but also on confidence in the technologies through which care is delivered.

Taken together, the five TRUST domains reinforce one another: workforce preparedness supports governance, governance shapes user engagement, engagement influences safety, and all four depend on trustworthy technologies and data practices. Collectively, these domains provide an integrated practice-based framework for strengthening trust, accountability, and quality within digital care contexts.

## 7 TRUST comparison with existing frameworks

The TRUST framework should be understood as complementary to, rather than a replacement for, established implementation science and digital health frameworks. Frameworks such as CFIR, NASSS, RE-AIM/PRISM, and TAM provide important insights into implementation determinants, technology adoption, sustainability, contextual fit, and user acceptance (16–20). These frameworks have made substantial contributions to understanding why digital health interventions succeed, fail, spread, or become sustained within healthcare systems.

However, the evidence synthesized in this review suggests that digital health transformation is not only an implementation challenge but also a practice challenge. Consistent with the practice-based perspective underpinning this manuscript, digital health outcomes are shaped by how healthcare workers, patients, informal e-providers, technologies, data systems, and governance arrangements interact within everyday healthcare activities. These interactions include communication, documentation, prescribing, referral, supervision, patient engagement, privacy protection, use of digital tools, and accountability for clinical decisions. While existing implementation frameworks acknowledge contextual and behavioural influences, they generally provide limited explicit attention to these situated digital health work practices, particularly in settings where formal health systems increasingly coexist with informal digital consultations, social-media-mediated health advice, e-patients, and emerging e-provider practices.

The TRUST framework therefore adds a practice-based layer to existing implementation science approaches. Rather than focusing primarily on determinants of implementation or technology acceptance, TRUST focuses on how accountable digital health work is enacted in everyday practice. It brings attention to workforce preparedness, provider legitimacy, user engagement, patient safety, stewardship, trusted technologies, confidentiality, and accountability within rapidly evolving digital and virtual care environments. In this sense, TRUST seeks to bridge implementation science, digital health governance, and practice-based scholarship by providing a context-sensitive framework for strengthening trustworthy digital health work practices in Tanzania and similar settings.

Table 3 compares TRUST with existing implementation science and digital health frameworks, highlighting both their strengths and the additional practice-based contribution offered by TRUST.

**Table 3:** Comparison of TRUST and existing implementation science frameworks.

Framework	Focus	Relevance to manuscript	Limitations/what TRUST adds
CFIR (16)	Multilevel determinants of implementation across intervention, inner setting, outer setting, individuals, and implementation process.	Useful for identifying factors influencing success or failure of digital health implementation across health systems and organizations.	Provides limited explicit attention to how digital health work is enacted in everyday practice. Does not specifically address provider verification, informal digital care pathways, patient trust, digital professionalism, stewardship, or accountability within virtual care interactions. TRUST adds a practice-based focus on how healthcare workers, patients, technologies, and governance mechanisms interact in digitally mediated care.
NASSS (17)	Non-adoption, abandonment, scale-up, spread, and sustainability of technology-supported care.	Highly relevant for understanding complexity, adoption challenges, and sustainability of digital health innovations.	Focuses primarily on technology implementation and sustainability rather than everyday digital health work practices. TRUST adds attention to workforce behaviour, provider legitimacy, patient engagement, stewardship, confidentiality, and trust in contexts where formal and informal digital care coexist.
RE-AIM/PRISM (18, 19)	Reach, effectiveness, adoption, implementation, maintenance, and contextual fit of interventions.	Useful for planning, implementation, and evaluation of digital health programmes and policy interventions.	Strong on programme evaluation but less explicit regarding the practical realities of virtual care work, e-patient interactions, digital professionalism, and governance of everyday digital practices. TRUST adds a practice-based perspective focused on accountability, communication, referral, prescribing, supervision, and trust within digitally mediated care.
TAM (20)	Technology acceptance based on perceived usefulness and perceived ease of use.	Useful for understanding patient and provider acceptance of digital tools and technologies.	Focuses on user acceptance rather than clinical governance, patient safety, stewardship, confidentiality, professional accountability, or digital health work practices. TRUST extends beyond acceptance to address how digital care is safely and responsibly enacted in practice.
TRUST	Transparent credentials and workforce preparedness; Robust clinical governance; User-centred engagement; Safety and stewardship; Trusted e-Health technologies and data practices.	Provides a practice-based, governance-oriented framework for accountable digital health work in environments where e-patients, e-providers, virtual care, and informal digital health practices increasingly coexist.	Proposed framework requiring empirical validation, implementation testing, and contextual adaptation. Its added contribution is the explicit focus on situated digital health work practices, workforce behaviour, provider legitimacy, patient safety, stewardship, trusted technologies, confidentiality, and trust within digital healthcare environments.

## 8 Recommendations for policy, curriculum, and digital health practice

The findings of this review and the proposed TRUST framework suggest that strengthening digital health in Tanzania requires more than technological expansion. Digital transformation should be accompanied by deliberate investments in workforce preparedness, governance, user engagement, stewardship, and trustworthy digital health practices. The following recommendations are proposed for policymakers, training institutions, professional councils, healthcare organizations, and digital health implementers.

### 8.1 Transparent training and credentials for digital health practice

Health professional education should evolve to reflect the realities of digitally mediated healthcare. Pre-service and in-service curricula should be strengthened to include competencies related to e-patients, e-providers, e-Health technologies, virtual consultations, e-nursing care, digital professionalism, privacy and confidentiality, AI-supported clinical decision-making, misinformation management, digital documentation, safe e-prescribing, referral systems, and antimicrobial stewardship. Importantly, these competencies should be developed through practice-based strategies rather than classroom instruction alone. Simulation exercises supervised virtual consultations, digital case-based learning, interprofessional education, and mentorship programmes should be incorporated into health professional training. Professional councils and regulatory bodies should also establish competency standards and continuing professional development requirements for virtual care practice and digital professionalism. In addition, mechanisms for transparent provider verification should be strengthened through interoperable professional registries, digital credentialing systems, and publicly accessible verification platforms that allow patients to confirm provider identity, licensure status, and scope of practice before engaging in virtual care.

### 8.2 Robust clinical governance for virtual care

National digital health governance frameworks should be expanded to include explicit standards for virtual care delivery. This should include guidance on patient identification, triage, documentation, referral, escalation pathways, supervision, clinical audit, incident reporting, and quality improvement within digital and virtual care environments. Also, healthcare organizations should develop practical protocols for e-consultations, telemedicine services, e-nursing care, remote follow-up, and AI-assisted decision support. These protocols should clarify professional responsibilities, accountability mechanisms, documentation requirements, and referral thresholds for cases requiring physical assessment or higher levels of care. Most importantly, supportive supervision and routine audits of virtual care practices should become integral components of digital health implementation to ensure that technology-enabled care remains aligned with professional, ethical, and regulatory standards.

### 8.3 User-centred engagement and digital health literacy

Digital health services should be designed and evaluated with meaningful participation from patients, communities, healthcare workers, and regulators. Co-design approaches can improve usability, acceptability, trust, and responsiveness to local needs. Patient digital health literacy should be strengthened through community education programmes, trusted public-facing information platforms, and partnerships with professional associations and public health agencies. Such initiatives should help patients identify credible sources of information, recognize legitimate providers, understand privacy risks, and engage safely with digital health services. Additionally, healthcare workers should receive training in empathetic e-communication, shared decision-making, misinformation management, and respectful engagement with digitally informed patients. Establishing patient feedback mechanisms for virtual care services may further strengthen accountability and trust.

### 8.4 Safety and stewardship in digital and virtual care

Patient safety and antimicrobial stewardship should be embedded within all virtual care services. Digital platforms should incorporate evidence-based prescribing guidance, clinical decision-support tools, referral safeguards, and mechanisms for monitoring prescribing patterns and virtual care quality. Relatedly, national stewardship programmes should explicitly address e-prescribing, online medicine access, and digitally mediated healthcare interactions. Professional councils and regulators should develop standards for safe virtual prescribing, monitoring of virtual care practices, and management of clinical risks associated with remote care. Healthcare organizations should also implement incident-reporting systems, virtual care quality audits, and referral escalation mechanisms to ensure that patient safety remains central to digital transformation efforts.

### 8.5 Trusted e-health technologies and data practices

Digital health investments should prioritize technologies that are secure, interoperable, user-friendly, and aligned with national governance frameworks. Strengthening interoperability remains essential for continuity of care, accountability, and efficient information exchange across healthcare levels. Organizations should establish clear guidance regarding consent procedures, authentication mechanisms, role-based access controls, secure communication channels, data storage practices, use of personal devices, and management of screenshots and shared information within virtual care environments. Attention should be given to privacy protection practices among both e-patients and informal e-providers.

Routine monitoring of cybersecurity risks, audit trails, breach-reporting mechanisms, and compliance with the Personal Data Protection Act should become integral components of digital health governance. Public awareness efforts should also improve understanding of patient rights related to privacy, confidentiality, and responsible use of digital health technologies.

### 8.6 Future research and implementation priorities

Future research should move beyond documenting digital health adoption and focus on understanding how digital health practices are enacted in real-world healthcare environments. Priority areas include empirical assessment of e-patient behaviours, informal digital consultations, e-provider practices, virtual care quality, digital professionalism, privacy and confidentiality practices, and patient trust within digitally mediated care environments. Research is also needed to evaluate healthcare workers' preparedness for virtual care, AI-supported decision-making, e-consultations, and e-nursing care, as well as the effectiveness of regulatory, credential-verification, stewardship, and governance interventions designed to strengthen accountability in digital health systems. Attention should be given to implementation and longitudinal studies that examine how virtual care integration influences continuity of care, prescribing practices, workforce adaptation, patient safety, and trust over time. Finally, the proposed TRUST framework requires empirical validation across diverse healthcare settings to assess its usefulness in strengthening accountable digital health work practices in Tanzania and similar low- and middle-income country contexts.

## 9 Limitations and delimitations

Several limitations and delimitations should be considered when interpreting the findings of this review. First, this article was intentionally designed as a narrative and policy-oriented synthesis rather than a formal systematic review or meta-analysis. The objective was not to exhaustively retrieve all digital health, telemedicine, virtual care, or e-patient literature related to Tanzania, but rather to synthesize selected policy documents, implementation reports, empirical studies, and conceptual literature relevant to workforce preparedness, governance, trust, confidentiality, patient safety, and stewardship within evolving digital health environments. Consequently, although elements of PRISMA-informed reporting were incorporated to strengthen transparency, the review should be interpreted as an implementation-oriented synthesis rather than a comprehensive evidence review.

Second, the available evidence base remains heterogeneous in scope, methodology, and depth. Much of the Tanzania-specific evidence on digital health focuses on governance reforms, interoperability, health information systems, referral coordination, and pilot implementation programmes, while comparatively fewer studies directly examine e-patient behaviour, informal digital consultations, patient trust, digital professionalism, or virtual care legitimacy. As a result, some sections of this review necessarily draw on broader conceptual literature, global implementation science frameworks, WHO guidance documents, and comparable evidence from sub-Saharan Africa to support contextual interpretation of emerging trends relevant to Tanzania. Several conclusions regarding informal digital consultations, patient trust dynamics, and digitally mediated healthcare behaviour should therefore be interpreted as theoretically and contextually informed inferences rather than directly demonstrated causal relationships.

Third, the review does not quantify the prevalence, frequency, or population-level characteristics of informal digital consultations, social-media-mediated prescribing, or use of non-credentialed e-providers in Tanzania. Similarly, this synthesis does not empirically measure patient trust, confidentiality perceptions, treatment outcomes, or long-term effects of virtual care integration. The review instead focuses on identifying emerging implementation, governance, and workforce issues that may become increasingly important as digital and virtual care ecosystems continue to expand. In this sense, the manuscript is intentionally forward-looking and interpretive rather than a predictive or epidemiological study.

Furthermore, although TRUST is informed by practice-based scholarship, the framework has not yet been empirically evaluated to determine whether the proposed domains adequately capture the full range of digital health work practices performed by healthcare workers, informal e-providers, and e-patients across different healthcare contexts. The framework was developed abductively through iterative synthesis of recurring workforce, governance, stewardship, confidentiality, and trust-related themes identified across the reviewed literature. Although TRUST is grounded in Tanzania-relevant evidence and broader implementation science literature, it has not undergone formal empirical validation, stakeholder consensus development, implementation testing, or comparative evaluation across healthcare settings. The framework should therefore be viewed as a practical organizing approach intended to stimulate further discussion, policy reflection, workforce planning, and future implementation research rather than as a finalized or universally generalizable model.

Despite these limitations, this synthesis contributes important value as an implementation-oriented analytical lens. The synthesis brings together evidence from governance reforms, health information systems, AI-supported diagnostics, virtual-care initiatives, antimicrobial stewardship literature, and emerging discussions on e-patients and informal digital consultations. This allowed the review to highlight how digital health transformation is increasingly intertwined with workforce preparedness, legitimacy, patient safety, confidentiality, and public trust. Importantly, the review also demonstrates that digital transformation in Tanzania is no longer solely a question of technological expansion, but increasingly a question of how digital systems is governed, interpreted, trusted, and integrated into everyday clinical and community healthcare interactions.

## 10 Conclusion

Tanzania's experience illustrates a digital health paradox: the same technologies that can improve access, continuity of care, and diagnostic efficiency can also undermine trust when they enable unregulated care, misinformation, unsafe prescribing, confidentiality breaches, and poorly governed digital interactions. National digital health strategies and enterprise architecture initiatives demonstrate that Tanzania is making important progress toward coordination, interoperability, and system integration (4, 6, 7). However, evidence from facility-level implementation consistently shows that workforce preparedness, usability challenges, infrastructure reliability, and integration into routine clinical workflows remain decisive determinants of success (32, 34–36).

The growing presence of e-patients and informal e-providers should not be viewed solely as a regulatory threat, but also as a signal that communities are seeking more responsive, respectful, and accessible healthcare services. The challenge for Tanzania and similar low- and middle-income countries is therefore not whether digital health should expand, but how it can expand safely, equitably, and credibly within environments where formal and informal systems coexist.

To address this tension, Tanzania and similar settings should prioritize several foundational actions before large-scale digital expansion. These include establishing verified provider identities within virtual care systems; strengthening clinical governance, referral, and escalation pathways; preparing the healthcare workforce for digital communication and AI-supported clinical decision-making; implementing secure and accountable data practices; and strengthening regulation against unsafe digital medical advice, informal prescribing, and unauthorized online medicine sales. These priorities are essential for maintaining public trust while ensuring that digital innovation supports, rather than weakens, healthcare quality and patient safety.

The TRUST framework offers a practical direction for achieving this balance. Rather than resisting digital transformation, the framework emphasizes shaping it through coordinated health and education sector interventions, workforce readiness, governance and regulatory strengthening, patient engagement, and trusted data stewardship (1, 2, 54, 55). As a conceptual and practice-based framework, TRUST provides a pragmatic approach for protecting medical authenticity and strengthening trust while scaling digital health and virtual care in Tanzania and comparable contexts.
